# Task-induced subjective fatigue and resting-state striatal connectivity following traumatic brain injury

**DOI:** 10.1016/j.nicl.2022.102936

**Published:** 2022-01-04

**Authors:** J. Bruijel, C.W.E.M. Quaedflieg, T. Otto, V. van de Ven, S.Z. Stapert, C. van Heugten, A. Vermeeren

**Affiliations:** a Dept of Neuropsychology & Psychopharmacology, Faculty of Psychology and Neuroscience, Maastricht University, Maastricht, the Netherlands; b Limburg Brain Injury Centre, Limburg, the Netherlands; c Dept of Work and Social Psychology, Faculty of Psychology and Neuroscience, Maastricht University, Maastricht, the Netherlands; d Dept of Cognitive Neuroscience, Faculty of Psychology and Neuroscience, Maastricht University, Maastricht, the Netherlands; e Dept of Medical Psychology, Zuyderland Medical Centre, Sittard-Geleen, the Netherlands; f School for Mental Health and Neuroscience, Dept of Psychiatry and Neuropsychology, Faculty of Health, Medicine and Life Sciences, Maastricht University Medical Center, Maastricht, Netherlands

**Keywords:** DMN, default mode network, EPI, Echo-Planar Imaging, FC, functional connectivity, HC, healthy controls, mPFC, medial prefrontal cortex, MRI, magnetic resonance imaging, PCC, posterior cingulate cortex, ROI, region of interest, RSME, Rating Scale for Mental Effort, rsFC, resting-state functional connectivity, rs-fMRI, resting-state functional magnetic resonance imaging, TBI, traumatic brain injury, VAS-f, visual analogue scale for fatigue, Traumatic brain injury (TBI), Fatigue, Resting-state fMRI, Functional connectivity, Striatum, Default mode network (DMN)

## Abstract

**Background:**

People with traumatic brain injury (TBI) often experience fatigue, but an understanding of the neural underpinnings of fatigue following TBI is still lacking. This study used resting-state functional magnetic resonance imaging (rs-fMRI) to examine associations between functional connectivity (FC) changes and task-induced changes in subjective fatigue in people with moderate-severe TBI.

**Methods:**

Sixteen people with moderate-severe TBI and 17 matched healthy controls (HC) performed an adaptive N-back task (working memory task) to induce cognitive fatigue. Before and after the task they rated their state fatigue level and underwent rs-fMRI. Seed-to-voxel analyses with seeds in areas involved in cognitive fatigue, namely the striatum and default mode network (DMN) including, medial prefrontal cortex and posterior cingulate cortex, were performed.

**Results:**

The adaptive N-back task was effective in inducing fatigue in both groups. Subjective task-induced fatigue was positively associated with FC between striatum and precuneus in people with TBI, while there was a negative association in HC. In contrast, subjective task-induced fatigue was negatively associated with FC between striatum and cerebellum in the TBI group, while there was no association in HC. Similar associations between task-induced subjective fatigue and DMN FC were found across the groups.

**Conclusions:**

Our results suggest that the subjective experience of fatigue was linked to DMN connectivity in both groups and was differently associated with striatal connectivity in people with moderate-severe TBI compared to HC. Defining fatigue-induced neuronal network changes is pertinent to the development of treatments that target abnormal neuronal activity after TBI.

## Highlights

• Fatigue is a very frequent and disabling symptom in traumatic brain injury (TBI).
• Effects of task-induced fatigue on resting-state functional connectivity (rsFC).
• Striatal rsFC relates differently to subjective fatigue in TBI compared to controls.
• Default mode network rsFC relates similar to subjective fatigue in TBI and controls.

## 1 Introduction

Fatigue is one of the most reported symptoms following traumatic brain injury (TBI; Beaulieu-Bonneau and Ouellet, 2017, Mollayeva et al., 2014). These feelings may negatively affect daily living and quality of life (Ponsford and Sinclair, 2014). Particularly, cognitive fatigue, - a subjective feeling of difficulty in continuing or starting mental tasks - is a dominating factor that limits people with TBI to lead a normal life including work and social activities (Johansson and Rönnbäck, 2014, Wylie and Flashman, 2017, Kluger et al., 2013). Following TBI diffuse axonal injury may occur, next to bleeding and bruising of the brain, resulting from the fast accelerating or decelerating forces to the head, causing shearing of long axon connections within the brain (Smith et al., 2003). Consequently, this might lead to disturbances in structural and functional connectivity (FC) of the brain’s networks (Sharp et al., 2014). Examining FC might thus be relevant to explore specific brain networks and mechanisms implicated in fatigue following TBI.

Cognitive fatigue, which results from prolonged periods or strenuous cognitive activity, has been suggested to be a signal generated by the brain when performance outcome no longer merits the effort required (Dobryakova et al., 2013, Kurzban et al., 2013). The basal ganglia-cortical loops, that involve motivational processing to exert effort, have been implicated in fatigue (Chaudhuri and Behan, 2000). Task-based neuroimaging studies indicate the basal ganglia as a central region for fatigue in both healthy participants (Wylie et al., 2020, Müller and Apps, 2019) and people with TBI (Wylie et al., 2017, Dobryakova et al., 2020). Furthermore, in TBI, increased brain activity in the basal ganglia, specifically in the striatum, has been implicated in fatigue both when measured using subjective ratings (Wylie et al., 2017, Dobryakova et al., 2020) and as an assumed result of task performance (Kohl et al., 2009, McAllister et al., 1999). However, whether TBI affects connectivity between the striatum and the rest of the brain and whether this is associated with the subjective experience of fatigue following TBI has not yet been examined.

Connectivity alterations at rest following fatigue and its relationship to subjective fatigue measures are particularly intriguing in light of the large impact fatigue has on the lives of people with TBI (Ponsford and Sinclair, 2014). Resting-state functional connectivity (rsFC) parameters are not related to a task (Fox and Raichle, 2007), making it possible to explore the diffuse effects of fatigue on the brain and potential biomarkers for fatigue. Especially, rsFC within the Default Mode Network (DMN) has been implicated in fatigue in multiple clinical populations including people with mild TBI (Zhou et al., 2012), multiple sclerosis (Høgestøl et al., 2019); and cancer (Hampson et al., 2015). Following moderate-severe TBI, both enhanced (Palacios et al., 2013, Shumskaya et al., 2017) and reduced (Arenivas et al., 2014) rsFC within the DMN have been reported. Furthermore, rsFC is known to be altered by earlier brain states (Grigg et al., 2010) and changes in rsFC following prolonged task performance have been associated with cognitive fatigue in healthy participants (Esposito et al., 2014) and people with mild TBI (Nordin et al., 2016). In addition, rsFC within the basal ganglia network has been implicated in cognitive fatigue in people with mild TBI (Nordin et al., 2016). However, rsFC changes associated with task-induced changes in subjective fatigue have not yet been examined in people with moderate-severe TBI.

The aim of this exploratory study was therefore to compare fatigue-induced changes in rsFC in the striatum and DMN between people with moderate-severe TBI and healthy controls (HC). To examine this, resting-state functional magnetic resonance imaging (rs-fMRI) measurements and subjective fatigue were assessed before and after participants performed a cognitively fatiguing task. This cognitive task, the adaptive N-back task, adjusts workload to the performance of the person, and thereby allows people with TBI and HC to invest comparable amounts of effort, and induce cognitive fatigue in both groups (Jaeggi et al., 2010). Similar levels of task-induced subjective fatigue in both groups allows us to examine changes in rsFC (comparing post- to pre-task rsFC) with task-induced fatigue between the groups. Previous studies indicated the striatum as an important area for fatigue during task performance in people with moderate-severe TBI (Wylie et al., 2017, Dobryakova et al., 2020, Kohl et al., 2009). In addition, rsFC of the basal ganglia and the DMN have been associated with fatigue in people with mild TBI (Zhou et al., 2012, Nordin et al., 2016). Based on these findings, it was hypothesized that the association between task-induced subjective fatigue and pre to post-task changes in rsFC of the striatum and DMN would be different in people with moderate-severe TBI compared to HC.

## 2 Methods and materials

### 2.1 Participants

Participants were right-handed individuals with a history of moderate-severe TBI, aged between 18 and 70 years, and age, sex, and education matched HC. Participants were recruited through advertisement, via an institutional register of prior study participants who gave permission to be contacted again for research, and via an outpatient rehabilitation unit at Zuyderland Medical center, where a neurologist or neuropsychologist confirmed the TBI and referred eligible candidates to the study. The Mayo classification system was used to confirm the TBI as moderate-severe using imaging data and/or injury characteristics including loss of consciousness, post-traumatic amnesia, and behavioural symptoms (Malec et al., 2007). Inclusion criteria for the TBI group were a history of moderate-severe TBI, completion of medical treatment for consequences of the TBI, and a time since injury between 6 months and 6 years so the majority of recovery already took place but participants were not too long since injury (Karr et al., 2014). HC needed to be in good self-reported overall health. For both groups, exclusion criteria were a history of neurological disorders (other than TBI), current medical disorders or treatment (including medication that could account for fatigue), a current diagnosed mental disorder based on clinical judgment and self-report, and contra-indications for the MRI scanner. Participants were recruited between June 2019 and August 2020.

### 2.2 Design and procedure

Participation consisted of two visits. During the first visit, participants signed the informed consent form, after which they were screened for contra-indications for scanning. Their general feelings of fatigue, anxiety, depression, and sleep quality over the past weeks were assessed by questionnaires (i.e. the Fatigue Severity Scale (Krupp et al., 1989), Hospital Anxiety and Depression Scale (Zigmond and Snaith, 1983), and Pittsburgh Sleep Quality Index (Buysse et al., 1989), respectively). Finally, participants were familiarized with the N-back task and scanning procedures by practicing the N-back task using an MRI-compatible optical 2-button joystick (Current Designs Inc., Philadelphia, USA), both outside and inside a mock scanner.

During the second visit, participants went in the MRI-scanner. First, an anatomical scan was made, followed by the first resting-state scan. Next, to induce fatigue participants were required to complete an adaptive N-back task in the scanner, which was followed by a second resting-state scan. Participants rated their state (momentary) fatigue level using the visual analogue scale for fatigue (VAS-f; LaChapelle and Finlayson, 1998) at four time points: at the start of the first resting-state scan (t0), before (t1) and after the task (t2), and at the end of the second resting-state scan (t3). At t2, participants also rated the amount of effort invested in the task using the Rating Scale for Mental Effort (RSME; Zijlstra, 1995). There was a minimum of 1 day and a maximum of 14 days between the visits. Visits were schedule according to the participants’ availability between 8:30am and 5 pm. Participants were requested to be well rested and to not consume caffeine in the hour before the visits. The study was carried out in accordance with The Code of Ethics of the World Medical Association (Declaration of Helsinki) for experiments involving humans and the study protocol was approved by the Ethics Review Committee Psychology and Neuroscience of Maastricht University (ERCPN- 198_12_09_2018).

### 2.3 Subjective rating scales of state fatigue and effort

State fatigue levels were assessed using the VAS-f (LaChapelle and Finlayson, 1998). The VAS-f consists of a horizontal line presented on a computer screen with the left end representing ‘absolutely no fatigue’ and the right end ‘most severe fatigue imaginable’ with no intermediate divisions or descriptive terms. Scores range from 0 to 100, with higher scores indicating more fatigue. The VAS-f was found to be valid and reliable and has been used in previous studies including people with TBI (LaChapelle and Finlayson, 1998, Bushnik et al., 2008). Task-induced subjective fatigue scores were calculated by subtracting fatigue levels pre-task (t1) from post-task (t2).

The RSME was used to assess subjective mental effort invested in the adaptive N-back task (Zijlstra, 1995, Widyanti et al., 2013). This scale was presented on a computer screen and consists of a vertical visual analogue scale ranging from 0 to 150 with nine anchor points with descriptive labels ranging from “absolutely no effort” (near 0 on the scale), through “rather much effort” (at 57 on the scale) to “extreme effort” (at 112 on the scale). Participants were requested to mark the line at the point corresponding to the amount of mental effort it took to complete the adaptive N-back task.

### 2.4 Adaptive N-back task

The adaptive N-back task is a working memory task measuring accuracy and reaction time and was adjusted from a dual N-back task of Jaeggi and colleagues (Jaeggi et al., 2008). Participants were shown a sequence of letters, one at a time, and had to respond with the trigger of the joystick (index finger) each time the current letter was identical to the one presented n positions back in the sequences and with the button of the joystick (thumb) if the letter was not identical. Stimuli were presented for 1000 ms and participants had 2000 ms to respond. The inter stimulus interval was jittered and was between 2000 ms and 4000 ms. The level of the n-back depended on the performance of the participant and could be 1-, 2-, 3-, and 4-back. Performance accuracy was calculated per round as the percentage of correct responses of the total number of trials. Missed responses were treated as an incorrect response. The average accuracy of the last two rounds was used to determine whether the level would change. When the participant had an accuracy of 90% or more, the level increased by one (to a maximum 4-back). When accuracy was 75% or less the level was decreased by one (to a minimum of 1-back). In all other cases, the level stayed the same. Participants did not receive feedback. Task duration was approximately 25 min. Participants were not informed that changes in n-back levels depended on their performance. By adjusting the difficulty of the task based on performance of the participants, we aimed to have high and similar amounts of effort invested by all participants and thereby induce fatigue.

### 2.5 MRI acquisition

Resting-state data were acquired on a 3T MAGNETOM Prisma Fit scanner (Siemens AG, Erlangen, Germany) with a 32-channel head coil by using a whole-brain Echo-Planar Imaging (EPI) with the following parameters: TR = 2000 ms, TE = 30 ms, voxel size 2x2 mm^2^, slice thickness = 2 mm, FoV = 200 mm, flip angle = 77°, slice order interleaved, no gap, acceleration factor 2, number of slices 60 and number of volumes 350. Participants were instructed to relax and not engage in any specific mental activity while keeping their eyes open and looking at a black screen with a white cross. Two EPI-scans with opposite phase encoding using similar parameters consisting of 5 volumes were acquired after the 1st and before the 2nd resting-state scan. An anatomical image was obtained for each participant using a high-resolution structural T1-weighted image, a volumetric magnetization prepared rapid gradient echo (MPRAGE) sequence with the following parameters: TR = 2250 ms, TE = 2.21 ms, TI = 900 ms, voxel size 1x1x1 mm^3^, FoV = 256 mm, and flip angle = 9°.

### 2.6 MRI and fMRI data pre-processing

MRI and fMRI data pre-processing was done using the standardized pre-processing pipelines of the CONN toolbox (version 19b; Whitfield-Gabrieli and Nieto-Castanon, 2012) that uses the pre-processing steps from SPM (version 12). In addition, we used FSL-topup to create fieldmaps using the opposite phase-encoded scans, for Susceptibility Distortion Correction (unwarping; Andersson et al., 2003). Motion correction/realignment, slice-timing correction, co-registration, tissue-class segmentation, and MNI-normalization were performed. Further cleaning of functional timeseries included identification of scans with excessive head motion for scrubbing (outlier scans identified using a conservative setting: 95th percentile, global signal z-value threshold of 3 and subject motion threshold of 0.5 mm) and removal of confounding effects from the BOLD signal (using least squares linear regression) of white matter and cerebrospinal fluid time series (5 regressors each using CompCor technique; Behzadi et al., 2007), six motion parameters and their first-order derivatives. Images were spatially smoothed using a 6 mm full-width at half-maximum (FWHM) Gaussian kernel and temporally filtered using the default temporal bandpass filter of 0.008 to 0.09 Hz. Due to scrubbing, the final length of the first resting-state scan ranged between 10 and 12 min (mean: 11.2, SD: 0.5) and the second resting-state scan between 8 and 12 min (mean: 10.9, SD: 0.9), which is still well above the 5 min lower limit for adequate resting-state data (Power et al., 2015). There was no significant difference in the number of scrubbed volumes between the pre- and post-task resting-state scan (*p* = .27) or between the groups (*p* = .17*)*. There was also no difference in mean head motion between the groups (TBI mean: 0.12, SD: 0.05; HC mean: 0.13, SD: 0.04 mm; *p =* .89) but there was a trend towards higher maximum motion in the TBI group (TBI mean: 2.77, SD: 3.60; HC mean: 0.87, SD: 0.64 mm: *p =* .053). Subjects with the most movement were not excluded since this might bias the sample (Wylie et al., 2014).

### 2.7 Statistical analysis

Statistical analyses of behavioral data were performed in R (version 3.5.1; R_Core_Team, 2014) and figures were made using the *ggplot2* package (Wickham, 2016). Statistical analyses of the rs-fMRI data were performed with the CONN toolbox (Whitfield-Gabrieli and Nieto-Castanon, 2012).

Independent sample t-tests and chi-square tests (or Welch *t*-test in case of unequal variances) were used to examine differences between the TBI and HC group in demographic and clinical characteristics. To examine whether the adaptive N-back task induced a similar increase in state fatigue (VAS-f) in both groups, linear mixed-effect regression models using the *nlme* package were performed. This model included group and time as predictors, with subject as random intercept to cluster observations within a subject, and the interaction between group*time to assess group difference in the change in fatigue levels over time (Pinheiro et al., 2013). Independent sample t-tests were used to examine differences between the TBI and HC group in mental effort (RSME), task-induced subjective fatigue (VAS-f t2 – VAS-f t1), and adaptive N-back performance including accuracy, and percentage of trials and reaction time for each N-back level.

For the resting-state data, seed-to-voxel analyses were performed. The ventral striatum was based on previous studies (Wylie et al., 2020, Dobryakova et al., 2020, Chen et al., 2020) assessing fatigue (MNI, 18, 12, 0, 512 mm^3^. The core seeds of the DMN including the medial prefrontal cortex (mPFC; MNI, 1, 55, −3, 10768 mm^3^) and posterior cingulate cortex (PCC; MNI, 1, −61, 38, 38664 mm^3^) were selected using the default network map of CONN, which is based on CONN's ICA analyses of Human Connectome Project dataset in 497 subjects (Whitfield-Gabrieli and Nieto-Castanon, 2012). The pre-processed time-series of each voxel within the seed were averaged. Seed-based connectivity maps were computed for each participant by calculating the bivariate correlation coefficient between the time-series of each seed and every voxel in the brain and transformed into Fisher-z correlation coefficients. For the rsFC analyses, group was included as between-subject factor (TBI, HC), time as within-subject factor (pre-, post-task), and sex and age (demeaned) were entered as covariates. First, pre-task rsFC was compared between the TBI and HC group using a one-way ANCOVA. The relationship between pre-task rsFC differences and state fatigue (VAS-f t0) was examined using multiple linear regression analyses. Second, it was investigated whether task-induced fatigue (VAS-f t2 – VAS-f t1) was associated with changes in rsFC (post – pre-task) over all subjects using multiple regression analysis. Third, associations between task-induced fatigue and changes in rsFC (pre- and post-task contrast) were compared between people with TBI and HC using a two-way ANCOVA interaction analysis. Post-hoc analyses of the significant associations were performed for each group separately to examine the association between task-induced fatigue and changes in rsFC per group. All group-level results were thresholded at *p* < .001 uncorrected for voxel-level (height threshold) and *p* < .05, uncorrected for cluster-size within the CONN toolbox (Friston et al., 1994); and a Bonferroni multiple comparison correction was used to control for false positives due to using three seeds. Thus, two-tailed cluster-size *p-values* < 0.0167, after voxel-level correction, were considered statistically significant.

## 3 Results

### 3.1 Group characteristics

In total, 34 participants (17 with TBI and 17 matched HC) were included in the study. Data from one TBI participant was excluded from analyses due to protocol violation (use of sleep medication). One TBI participant experienced a sudden discomfort in the last four minutes of the post-task resting-state scan, so these minutes are missing but this participant was still included in all analyses. Demographic and clinical characteristics of each group are summarized in Table 1. Around half of the participants with TBI reported severe levels of fatigue (FSS ≥ 4). Trait fatigue levels (FSS), depression, and anxiety symptoms (HADS) were significantly higher in the TBI group compared to HC (Table 1). There were no significant group differences in age, sex, education, living situation, or sleep quality (PSQI). Hours of employment were lower in the TBI group compared to HC. In the TBI group, the causes of injury were motor vehicle accidents (n = 8), falls (n = 7), and assault (n = 1).

**Table 1:** Demographics and subjective symptomatology in the traumatic brain injury (TBI) and healthy control (HC) groups.

	TBI (N = 16)		HC (N = 17)		
	Mean ± SD or N (%)	Range	Mean ± SD or N (%)	Range	*p-*value
Age (years)	37.1 ± 13.6	21–65	39.0 ± 13.4	20–67	*t*(31) = 0.41, *p* = .68
Sex (male)	12 (75.0%)		13 (76.5%)		Χ^2^(1, 33) = 0.01, *p* = .92
Years of education	17.1 ± 2.7	11–21	16.8 ± 1.9	14–19	*t*(31) = −0.33, *p* = .74
Living independently	16 (100%)		17 (100%)		Χ^2^(1, 33) = 0.00, *p* = 1.0
Employed at the moment of the study	12 (75%)		14 (82.4%)		Χ^2^(1, 33) = 0.27, *p* = .61
Hours of employment (per week)	29.3 ± 10.0	8–40	35.9 ± 5.6	24–40	*t*(24) = 2.12, *p* = .045
Medication, yes	4 (29%)		4 (24%)		Χ^2^(1, 33) = 0.01, *p* = .92
Psycho-active medication	0 (0%)		0 (0%)		
Time since injury (months)	25 ± 2.2	6–71	–	–	
Post-traumatic amnesia (days)	8.7 ± 11.9	0–30	–	–	
Loss of consciousness (hours)	49 ± 99.9	0–336	–	–	
Start time of the 2nd visit	12:20 ± 2:25	8:30–17:00	13:03 ± 2:29	9:00–17:00	*t*(31) = −0.83, *p* = .41
Sleep quality (PSQI score)	6.5 ± 3.4	1–13	4.8 ± 2.7	1–11	*t*(31) = −1.57, *p* = .13
*Clinically significant (>5*)	10 (63%)		5 (29%)		
Trait Fatigue (FSS score)	4.0 ± 1.5	1.4–6.3	3.1 ± 1.0	1.2–4.9	*t*(31) = −2.09, *p* = .045
*Clinically significant (*≥*4*)	8 (50%)		3 (18%)		
Depression (HADS score)	6.2 ± 4.4	1–14	2.5 ± 2.4	0–8	*t*(22.7) = −3.02, *p* = .006
*Clinically significant (*≥*8*)	7 (43.8%)		1 (6%)		
Anxiety (HADS score)	6.2 ± 2.4	2–10	3.4 ± 2.3	0–8	*t*(31) = −3.40, *p* = .002
*Clinically significant (*≥*8*)	5 (31.3%)		1 (6%)		
SD, standard deviation; PSQI, Pittsburgh Sleep Quality Index; FSS, Fatigue Severity Scale; HADS, Hospital Anxiety and Depression Scale.					

### 3.2 State fatigue, effort, and N-back performance

**Fig. 1:**
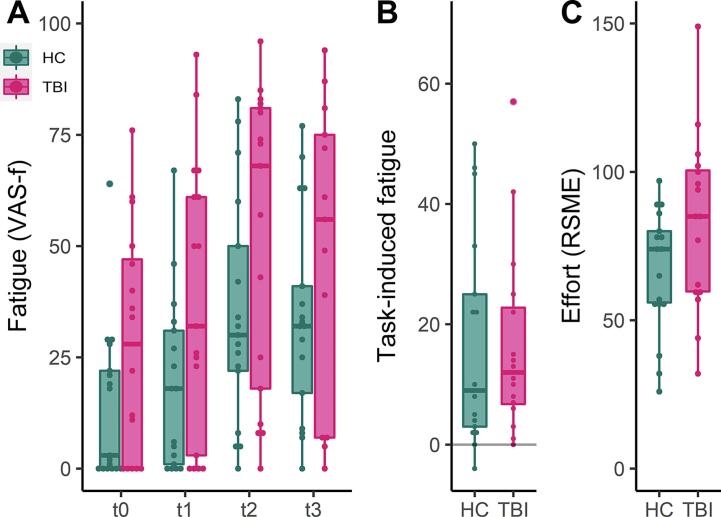
Individual subjective fatigue scores at each time point (A), task-induced subjective fatigue (B), and mental effort scores (C) of the traumatic brain injury (TBI) and healthy control (HC) group. The central marks in the boxplots present the median, the edges the 25th and 75th percentiles, and the lines extend to the most extreme data points not considering outliers, each participant is presented as a dot. VAS-f visual analogue scale for fatigue; RSME rating scale mental effort; t0 start of the first resting-state scan; t1 before the task; t2 after the task (t2); t3 end of the second resting-state scan (t3).

Fatigue scores (VAS-f) at each time point, task-induced fatigue (VAS-f t2 –t1), and mental effort scores (RSME) are presented in Fig. 1. First, we determined whether the adaptive N-back task-induced fatigue in both groups. There was a significant effect of time indicating that fatigue levels increased over time in both groups (β = 9.42, 95%CI: 6.41 – 10.59, *p* < .001; Fig. 1a). There was also a significant effect of group, indicating that fatigue was significantly higher in the TBI group compared to the HC group (β = 9.10, 95%CI: 0.89 – 35.44, *p* = .04). As expected, there was no significant difference in the change of fatigue over time between people with TBI and HC (interaction group*time, β = 1.02, 95%CI: −2.37 – 6.04, *p* = .39). In people with TBI there was no correlation between depression/anxiety (HADS) and task-induced fatigue (Table S2).

Self-reported mental effort (RSME, *t*(31) = −1.82, *p* = .08; Fig. 1c) and task-induced subjective fatigue (*t*(31) = 0.15, *p* = .99; Fig. 1b) did not differ significantly between the groups. Overall percentage correct responses in the N-back was similar between groups (*t*(31) = 1.61, *p* = .12; Table S1). The adaptive nature of the N-bask task resulted in a higher proportion of trials on the less demanding 1-back level (Welch *t*(16.39) = −2.18, *p* = .044) in participants with TBI compared to HC (Table S1).

### 3.3 Baseline rsFC

First, we examined baseline differences in rsFC between the TBI and HC group (Table S3). Pre-task rsFC differences were present in the mPFC, PCC, and striatum. rsFC was enhanced in the TBI group compared to HC between the mPFC and left angular gyrus, left supramarginal gyrus, and left inferior frontal gyrus. In contrast, rsFC was reduced between the PCC and left middle temporal gyrus and between the striatum and the left occipital fusiform gyrus in the TBI group compared to the HC group. Importantly, the differences in rsFC between the groups were not associated with state fatigue at t0 (all *p*’s > 0.31).

### 3.4 Task-induced fatigue associated with rsFC

**Fig. 2:**
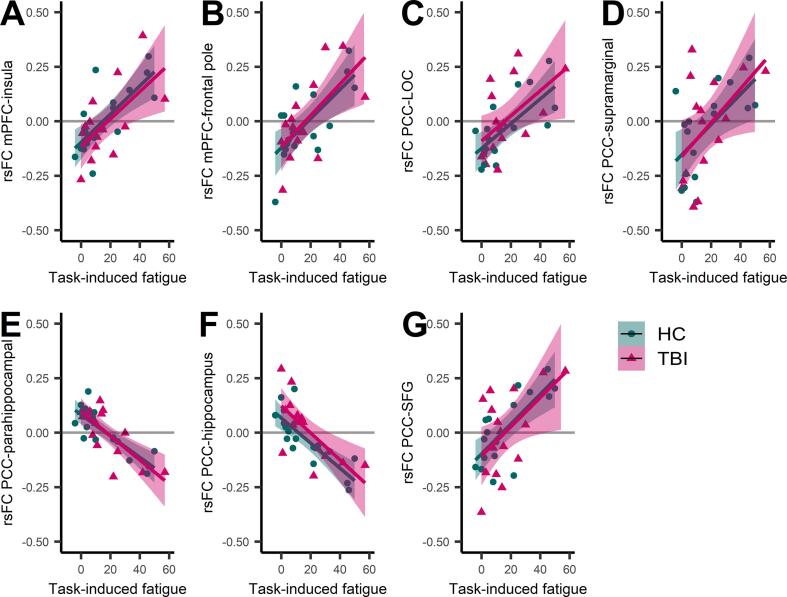
Associations between changes in resting-state functional connectivity (rsFC) with seeds in the default mode network and task-induced subjective fatigue over all subjects. As shown, the associations between rsFC changes (post – pre-task) and task-induced subjective fatigue are in the same direction for both the traumatic brain injury (TBI) and healthy control (HC) group indicating that not one of the groups is driving the associations.mPFC, medial prefrontal cortex; PCC, poster cingulate cortex; LOC, lateral occipital cortex; SFG, superior frontal gyrus.

Second, we investigated whether task-induced changes in fatigue were associated with changes in rsFC (post – pre-task) over all participants (Table 2). Increased task-induced fatigue was (positively) associated with enhanced rsFC between the mPFC and insula and frontal pole, and between the PCC and lateral occipital cortex, supramarginal gyrus, and superior frontal gyrus. Further, increased task-induced subjective fatigue was negatively associated with rsFC between the PCC and parahippocampal gyrus and hippocampus. These associations between task-induced subjective fatigue and rsFC changes were in the same direction for both the TBI and HC group indicating that these associations were not driven by one of the groups (Fig. 2).

**Table 2:** Resting-state functional connectivity associated with fatigue in people with traumatic brain injury (TBI) and healthy controls (HC). (Upper panel) regions with a significant association between task-induced fatigue and changes in rsFC (post – pre-task) over all participants and (lower panel) regions where the associations were significantly different in people with TBI compared to HC.

		Size (voxels)	Coordinates			F-stats (df) model						
**All subjects**	L/R		x	y	z	T(2, 29)	*p*-voxel	*p*-cluster				
*Medial prefrontal cortex*												
Insular cortex	L	58	–32	20	−2	6.19	0.000000	0.002847				
Frontal pole	R	42	22	48	−14	5.04	0.000056	0.008855				
*Posterior cingulate cortex*												
Lateral occipital cortex inferior division	L	127	−52	−72	−10	5.92	0.000018	0.000053				
Supramarginal gyrus anterior division	L	87	−64	−28	30	4.52	0.000052	0.000473				
Parahippocampal gyrus anterior division hippocampus	R	84	22	−10	−24	−8.40	0.000001	0.000565				
Hippocampus Amygdala	L	57	–22	−10	−16	−6.25	0.000002	0.003103				
Superior frontal gyrus	L	55	−24	2	70	4.67	0.000199	0.003554				
**Differences TBI vs HC**									changes rsFC (post – pre)		T-stats (df) per group	
			x	y	z	F(1,27)	*p*-voxel	*p*-cluster	TBI	HC	TBI	HC
*Striatum*												
Precuneus cortex		66	−8	−70	+54	27.14	0.000011	0.000589	0.07	−0.07	t(12) = 3.30, *p* = .006	t(13) = − 3.99, *p* = .0015
Cerebellum 4,5	L	36	−4	−58	−18	37.46	0.000058	0.009234	0.04	0.02	t(12) = −7.61, *p* = .000006	t(13) = 1.68, *p* = .12
T(2,29) threshold > 3.66; F(1,27) threshold > 13.61; uncorrected voxel threshold *p* < .001; uncorrected cluster threshold *p* < .0167 (Bonferroni corrected). Analyses were controlled for age and sex. rsFC, resting-state functional connectivity (fisher-z transformed correlation coefficients).

**Fig. 3:**
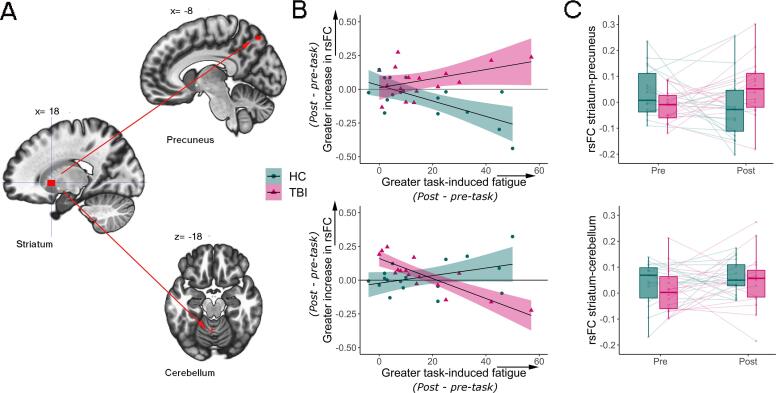
Striatum resting-state functional connectivity (rsFC) was differently associated with task-induced subjective fatigue in people with traumatic brain injury (TBI) compared to healthy controls (HC). **A & B**. Map of the associations between the striatal seed and precuneus (top part of the image) and, the striatal seed and cerebellum (bottom of the image), there was an opposite association between changes in rsFC and task-induced subjective fatigue in the TBI and HC group. B includes the 95% confidence interval. **C.** rsFC (Fisher z) between striatum and precuneus (top part of the image) and between striatum and cerebellum (bottom part of the image) at pre- and post-task for TBI and HC group. The central marks in the boxplots present the median, the edges the 25th and 75th percentiles and the vertical lines extend to the most extreme data points not considering outliers, each participant is presented as a dot and change in rsFC from pre to post-task for each participant is presented as a transparent line.

Third, we examined whether task-induced changes in fatigue were differently associated with changes in rsFC (post – pre-task) between the TBI and HC group. There was a significantly different association between task-induced fatigue and changes in rsFC for the striatum seed (Table 2; Fig. 3). Increased task-induced subjective fatigue was (positively) associated with enhanced rsFC between the striatum and precuneus extending to the left lateral occipital cortex in the TBI group but negatively associated with rsFC in the same areas in HC. In addition, participants with TBI showed an (negative) association between increased fatigue and reduced striatal-cerebellar rsFC, while this association was not significant in HC. For the seeds of the DMN, including the mPFC and PCC, task-induced fatigue was not differently associated with changes in rsFC in people with TBI compared to HC.

## 4 Discussion

We employed a version of the adaptive n-back task (Jaeggi et al., 2008) to induce similar increases in fatigue in people with moderate-severe TBI and HC. This allowed us to compare associations between task-induced subjective fatigue and changes in rsFC in the striatum and DMN between groups. The adaptive N-back induced a similar increase in fatigue in both groups. In neural terms, the two groups differed in striatal and DMN rsFC at baseline, but the differences were not associated with state fatigue at baseline. Further, both groups showed similar associations between task-induced subjective fatigue and pre- to post-task changes in rsFC of the mPFC and PCC. Together, these findings indicate that people with TBI and HC showed similar alterations in DMN connectivity with the experience of subjective fatigue while differing in striatal connectivity with the experience of fatigue.

Our finding of differential striatal connectivity following a fatiguing task extends on previous studies that only reported an association between fatigue and regional activity during task performance (Wylie et al., 2017, Dobryakova et al., 2020, Kohl et al., 2009). Specifically, in people with TBI, task-induced subjective fatigue was positively associated with rsFC between the striatum and precuneus, while increased task-induced subjective fatigue was associated with reduced rsFC in HC. This is in line with a study from Wylie and colleagues, which showed a positive association between fatigue and activity in the precuneus during task performance in people with TBI and a negative association in HC (Wylie et al., 2017). Furthermore, our results showed that in the TBI group, reduced rsFC between the striatum and cerebellum was associated with a larger increase in task-induced subjective fatigue, while there was no association in HC. The cerebellum may be involved in reward processing (Pierce and Péron, 2020) and an interconnected network between the basal ganglia, cerebellum and cerebral cortex has been proposed (Bostan and Strick, 2018). Given the role of dopamine in reward processing and a recent study in mice indicating a direct dopaminergic pathway between the basal ganglia and the cerebellum, our result might be in accordance with the dopamine imbalance hypothesis of fatigue. This hypothesis suggests that fatigue in neurological disorders originates from an imbalance in dopamine in the central nervous system (Dobryakova et al., 2015). These results contribute to the growing body of evidence indicating an association between structural (Clark et al., 2018) and functional changes in the striatum and fatigue in people with TBI (Wylie et al., 2017, Dobryakova et al., 2020, Kohl et al., 2009, Berginström et al., 2018). Moreover, our finding of altered resting-state striatal connectivity, even after task performance, and its association with subjective fatigue, highlights regional specificity in explaining TBI-related fatigue, and further suggests that striatal connectivity might be used as a neuronal correlate of fatigue following TBI. The involvement of the ventral striatum in motivational processing (Daniel and Pollmann, 2014) might contribute to evaluating if the effort needed to invest in a task is worth the outcome (Vassena et al., 2014), taking into account current levels of fatigue (Massar and Csathó, 2018, Müller et al., 2021). In other words, alterations in striatal connectivity in people with TBI might thus indicate a difference in the integration of fatigue and behavioral outcome and thereby influence the motivation to exert effort. However, it remains unknown whether TBI patients perceive effort differently, or whether they are less sensitive to outcome or reward value (Klein-Flugge et al., 2016). Future research could use varying feedback, effort and reward levels to evaluate this and explore the role of intrinsic and extrinsic motivational factors in fatigue in people with TBI.

In both groups, task-induced subjective fatigue was associated with enhanced rsFC between the medial DMN and widespread cortical areas and reduced rsFC between PCC and limbic areas. Based on the ample reporting of the DMN being associated with internal thoughts and mind-wandering (Mak et al., 2017, Mason et al., 2007), enhanced rsFC in our study between areas of the DMN and salience (insula) and executive control network (superior frontal gyrus, frontal pole) could indicate less internal focus and self- awareness in response to fatigue or fatigue-related compensatory connectivity in both groups (Clemens et al., 2017). People with TBI may experience high levels of fatigue on a daily basis and this might decrease their self-awareness. Future studies, could examine if people with TBI have trouble with awareness and how this relates to fatigue (Möller et al., 2017). The DMN might thus play a similar role in cognitive fatigue in people with TBI and HC.

Both groups reported investing similar amounts of effort to perform the task. Furthermore, state fatigue levels increased at a similar rate for both groups following task performance, indicating that changes in rsFC were not due to a difference in the fatigue-inducing effects of the adaptive N-back task. In general, state fatigue levels were higher in people with TBI compared to HC in our study. This is in line with the higher trait fatigue levels (FSS) found at the first visit in the TBI group compared to the HC group and with the findings of previous studies, which reported high levels of fatigue in people with TBI (Beaulieu-Bonneau and Ouellet, 2017, Mollayeva et al., 2014, Wylie et al., 2017). However, these higher state fatigue levels were not related to the baseline differences in rsFC of the DMN and striatum between people with TBI and HC. Results of previous studies have been inconsistent regarding an association between altered rsFC in the DMN in people with TBI compared to HC and behavior, such as impairments in cognition (Zhou et al., 2012, Shumskaya et al., 2017, Bonnelle et al., 2011, De Simoni et al., 2016). Our results indicate no association between altered baseline rsFC in the DMN or striatum and subjective fatigue in people with TBI.

Some limitations to this study should be mentioned. First, as often reported the pathology underlying a TBI is very heterogeneous, every brain injury is unique and has a complex interaction with multiple networks, which limits the generalizability of research findings. We tried to reduce heterogeneity by including patients in a stable time window post-injury (time post-injury between 6 months and 6 years) and by only including people with moderate-severe TBI. Second, the relatively broad time since injury and age range of patients in our study could pose limitations, given reports of age affecting FC in TBI and the risk of vascular events in elderly (Bittencourt-Villalpando et al., 2021, de Souza et al., 2020). However, we controlled for age in all our analyses. Furthermore, we did not check for sleep quality and sleep duration on the night preceding the examination, which could have affect fatigue levels. However, there was no differences in overall sleep quality (PSQI) between the groups. Due to the adaptive nature of the N-back task participants performed at different levels of the N-back task. Previous studies have shown that different N-back levels might affect neural activity (Lamichhane et al., 2020) and may thereby affect post-task rsFC. In our case, percentage of trials participants spent in the different N-back levels did not vary with differences in post-task rsFC between the groups, suggesting that the adaptive of the N-back did not confine the results. However, this is something to take into account for future studies using an adaptive N-back. Finally, sample size was relatively small, though it is comparable to other recent studies including participants with TBI with similar methodologies (Dobryakova et al., 2020, Nordin et al., 2016, Gilbert et al., 2018). rsFC changes in relation to fatigue have rarely been examined in people with moderate-severe TBI and further research with larger samples is necessary.

In conclusion, our results suggest a possible modulation in motivational processes, indicated by altered resting-state striatal functional connectivity, as an underpinning of cognitive fatigue in people with TBI, illustrating the importance of the connectivity of the striatum in the experience of fatigue following TBI. The mPFC and PCC of the DMN, on the other hand, did not show a different response to cognitive fatigue in people with TBI and HC. These areas of the DMN might therefore play a similar role in cognitive fatigue in people with TBI and HC. Further knowledge of striatal connectivity as a neural correlate of fatigue could increase our understanding of the mechanisms behind fatigue in people with TBI and maybe assist in the diagnosis of fatigue. These findings might contribute to the development of treatments for fatigue following TBI aimed at abnormal striatal connectivity.

### CRediT authorship contribution statement

**J. Bruijel:** Conceptualization, Data curation, Formal analysis, Investigation, Writing – original draft, Visualization, Project administration, Writing – review & editing. **C.W.E.M. Quaedflieg:** Visualization, Methodology, Writing – review & editing. **T. Otto:** Conceptualization, Methodology, Writing – review & editing. **V. van de Ven:** Methodology, Writing – review & editing. **S.Z. Stapert:** Conceptualization, Writing – review & editing. **C. van Heugten:** Conceptualization, Writing – review & editing. **A. Vermeeren:** Conceptualization, Writing – review & editing.

## Declaration of Competing Interest

The authors declare that they have no known competing financial interests or personal relationships that could have appeared to influence the work reported in this paper.
