# Supplementary material for: Task-induced subjective fatigue and resting-state striatal connectivity following traumatic brain injury
Source: Neuroimage Clin. 2022 Jan 4;33:102936. doi: 10.1016/j.nicl.2022.102936 (PMC8749448; doi:10.1016/j.nicl.2022.102936)
Supplement: Supplementary data 1 [file mmc1.docx]

## Supplementary materials

### Results N-back

The adaptive nature of the N-back task resulted in a higher proportion of trials on the less demanding 1-back level (p= .044) in participants with TBI compared to controls. Conversely, for controls it resulted in a trend towards a higher proportion of trials on the more demanding 4-back level (p= .060) compared to the TBI group. Overall percentage correct responses in the n-back was similar between groups. Mean response times were comparable between the groups.

| **Table S1.** Performance of the adaptive N-back of participants with traumatic brain injury (TBI) and healthy controls (HC). | | | | | |
| --- | --- | --- | --- | --- | --- |
|  | TBI (N=16) | | HC (N=17) | |  |
|  | Mean | SD | Mean | SD | *p-*value |
| 1-back RT | 679.8 | 96.5 | 706.3 | 130.5 | t(31)=-0.7 *p*=.51 |
| 2-back RT | 813.9 | 166.2 | 823.8 | 157.7 | t(30)=-0.2 *p*=.86 |
| 3-back RT | 786.2 | 122.4 | 879.1 | 135.8 | t(27)=-1.9 *p*=.07 |
| 4-back RT | 798.6 | 118.5 | 811.2 | 103.9 | t(21)=-0.27 *p*=.79 |
| 1-back percentage | 26.3 | 30.2 | 9.4 | 6.7 | t(16.4)=2.2 *p*=.044 |
| 2-back percentage | 20.0 | 21.2 | 12.2 | 15.1 | t(27.0)=1.2 *p*=.23 |
| 3-back percentage | 18.8 | 21.3 | 19.6 | 23.5 | t(31)=-0.1 *p*=.91 |
| 4-back percentage | 35.0 | 37.2 | 58.8 | 32.8 | t(31)=-2.0 *p*=.060 |
| Accuracy complete task | 0.80 | 0.13 | 0.85 | 0.04 | t(31)=-1.6 *p*=.12 |
| RT, reaction time; SD, standard deviation. | | | | | |
