# Supplementary material for: Task-induced subjective fatigue and resting-state striatal connectivity following traumatic brain injury
Source: Neuroimage Clin. 2022 Jan 4;33:102936. doi: 10.1016/j.nicl.2022.102936 (PMC8749448; doi:10.1016/j.nicl.2022.102936)
Supplement: Supplementary data 2 [file mmc2.docx]

## Supplementary materials

| **Table S2**. Pearson correlation coefficient (r) between depression, anxiety and fatigue in people with TBI. | | | | | |
| --- | --- | --- | --- | --- | --- |
|  | VAS-f t1 | VAS-f t2 | Task-induced subjective fatigue | Sleep quality (PSQI) | Trait Fatigue (FSS) |
| Depression (HADS) | 0.26 | 0.34 | 0.20 | 0.57*** | 0.72*** |
| Anxiety (HADS) | 0.28 | 0.25 | 0.00 | 0.73*** | 0.53** |
| HADS, Hospital Anxiety and Depression Scale; VAS-f, Visual Analogue Scale for Fatigue; PSQI, Pittsburgh Sleep Quality Index; FSS, Fatigue Severity Index. *p<.05; ** p<.01; p<.001. | | | | | |
