# Supplementary material for: Task-induced subjective fatigue and resting-state striatal connectivity following traumatic brain injury
Source: Neuroimage Clin. 2022 Jan 4;33:102936. doi: 10.1016/j.nicl.2022.102936 (PMC8749448; doi:10.1016/j.nicl.2022.102936)
Supplement: Supplementary data 3 [file mmc3.docx]

## Supplementary materials

| **Table S3.** Regions with significant baseline rsFC differences between the traumatic brain injury (TBI) and healthy control (HC) group. | | | | | | | | | | |
| --- | --- | --- | --- | --- | --- | --- | --- | --- | --- | --- |
|  |  | Size (voxels) | Coordinates | | | F-stats (df) model | | | rsFC pre-task | |
| **Baseline resting-state** | L/R |  | x | y | z | F(1,29) | *p*-voxel | *p*-cluster | TBI | HC |
| **TBI > HC** |  |  |  |  |  |  |  |  |  |  |
| ***DMN***  *Medial prefrontal cortex* |  |  |  |  |  |  |  |  |  |  |
| Angular gyrus | L | 159 | -40 | -52 | +36 | 30.68 | .000011 | .000034 | 0.20 | -0.09 |
| Supramarginal Gyrus,  posterior division | L | 65 | -52 | -44 | +48 | 18.78 | .000125 | .003430 | 0.06 | -0.24 |
| Inferior Frontal Gyrus,  pars opercularis | L | 54 | -56 | +20 | +26 | 33.77 | .000010 | .006635 | 0.11 | -0.16 |
| **TBI < HC** |  |  |  |  |  |  |  |  |  |  |
| *Posterior cingulate cortex* |  |  |  |  |  |  |  |  |  |  |
| Middle Temporal Gyrus  anterior division | L | 96 | -66 | -6 | -10 | 36.34 | < .000001 | .000660 | -0.06 | 0.15 |
| *Striatum* |  |  |  |  |  |  |  |  |  |  |
| Occipital fusiform gyrus | L | 41 | -16 | -80 | -10 | 24.45 | .000104 | .006975 | -0.01 | 0.09 |
| F(1,29) threshold > 13.39; uncorrected voxel threshold *p* < .001; uncorrected cluster threshold *p* < .0167 (Bonferroni corrected). Analyses were controlled for age and gender. rsFC, resting-state functional connectivity (fisher-z transformed correlation coefficients). | | | | | | | | | | |
